# The Influence of Virtual Reality Head-Mounted Displays on Balance Outcomes and Training Paradigms: A Systematic Review

**DOI:** 10.3389/fspor.2020.531535

**Published:** 2021-02-09

**Authors:** Pooya Soltani, Renato Andrade

**Affiliations:** ^1^Department of Computer Science, Department of Health, Centre for the Analysis of Motion, Entertainment Research and Applications (CAMERA), University of Bath, Bath, United Kingdom; ^2^Department of Physical Education and Sport Sciences, School of Education and Psychology, Shiraz University, Shiraz, Iran; ^3^Clínica do Dragão, Espregueira-Mendes Sports Centre, FIFA Medical Centre of Excellence, Porto, Portugal; ^4^Dom Henrique Research Centre, Porto, Portugal; ^5^Faculty of Sport, University of Porto, Porto, Portugal

**Keywords:** head-mounted display (HMD), posture (MeSH), vestibular, somatosensory, visual, older adults, gender, balance

## Abstract

**Background:** Falls are the leading causes of (non)fatal injuries in older adults. Recent research has developed interventions that aim to improve balance in older adults using virtual reality (VR).

**Purpose:** We aimed to investigate the validity, reliability, safety, feasibility, and efficacy of head mounted display (HMD) systems for assessing and training balance in older adults.

**Methods:** We searched EBSCOhost, Scopus, Web of Science, and PubMed databases until 1 September 2020 to find studies that used HMD systems for assessing or training balance. The methodological quality was assessed using a modified version of Downs and Black. We also appraised the risk of bias using Risk of Bias Assessment tool for Non-randomized Studies (RoBANS).

**Results:** A total of 19 articles (637 participants) were included for review. Despite heterogenous age ranges and clinical conditions across studies, VR HMD systems were valid to assess balance and could be useful for fall prevention and for improving postural control and gait patterns. These systems also have the capacity to differentiate healthy and balance-impaired individuals. During VR versions of traditional balance tests, older adults generally acquire a cautious behavior and take more time to complete the tasks.

**Conclusion:** VR HMD systems can offer ecologically valid scenarios to assess and train functional balance and can be used alone or in addition to other interventions. New norms and protocols should be defined according to participants' age, health status, and severity of their illness when using VR HMD systems for balance assessment and training. For safe and feasible training, attention must be given to display type, VR elements and scenarios, duration of exposure, and system usability. Due to high risk of bias and overall poor quality of the studies, further research is needed on the effectiveness of HMD VR training in older adults.

## Introduction

Maintaining balance while standing and during gait is paramount because stability plays a crucial role in human locomotion (Wodarski et al., 2019). Balance is mostly achieved and maintained by sensorimotor control systems that include sensory inputs from vision, proprioceptive, and vestibular systems (Manchester et al., 1989). Vision uses the information projected on the retina to guide the relationship between the environment and the body. The proprioceptive sensation provides information on the position and joint movements. The vestibular system combines gravity and acceleration inputs to collect information on the position and movement of the head. Changes in any sensory source elicit alterations in postural control. Of the three systems, vision is often considered as the most important factor for maintaining balance during quiet standing and activity (Horiuchi et al., 2017). Vision consists of the optical flow and visual field (central and peripheral). Optical flow deals with the perception of self-motion, and provides information about heading direction (Gibson, 1950). In the peripheral visual field, optical flow also contributes to a better postural sway stabilization (Horiuchi et al., 2017).

Falls affect around one-third of older adults and often put their independent functionality at risk (Al-Aama, 2011). Balance assessment should be an integral part of any fall prevention program to establish the baseline levels and monitor progression. Several methods are being used to assess the risk of falls, including surveys, physical tests, and perturbation-based measurements. Surveys are useful to measure the external risk factors but should be complemented with physical tests for measuring internal risk factors. Questionnaires could also be influenced by the participants' age, sex, and motivation, as well as cognitive and emotional status (Yardley and Redfern, 2001). Physical test batteries are easy to administer but may lack ecological validity and the ability to isolate specific sensory impairments (Saldana et al., 2017). For example, sensory organization test (SOT) is considered as the gold standard in estimating sensory contributions to balance control, but is unable to diagnose clinical disorders, such as vestibular hypofunction, because the postural sway is not a good indicator of underlying pathology (Lubetzky et al., 2018). Other systems that evaluate the isolated role of vision on standing posture have also their limitations. For example, Prism glasses alter a view on sagittal and horizontal planes, but not on the coronal plane (Ohmura et al., 2017). Balance training interventions include various static and dynamic routines for overcoming daily tasks in older adults (Halvarsson et al., 2014). However, such interventions may produce mixed success results as their exercises may not target the neuromuscular skills required for preventing falls (Parijat et al., 2015). The rehabilitation programs are also time-consuming and often lack adherence and compliance (Meldrum et al., 2015). Alternative approaches are needed to customize the interventions to the participants' needs, to objectively measure performance, and to boost participants' motivation while improving the adherence and compliance (Avola et al., 2019).

With the advancement of technology, many researchers are exploring the use of active video games (exergames) and virtual reality (VR) as assessment and rehabilitation tools. These systems use computer interfaces to immerse participants in virtual worlds. VR allows investigating functional balance performance without the risks associated with the previous methodologies (Oddsson et al., 2007). Sensory domes and foams have been used to provide head-fixed visual references as well as inaccurate somatosensory feedback. Tilting floors and moving walls are also used to offer sensory destabilization in the sagittal plane resulting in increased sway in the anteroposterior direction (Alahmari et al., 2014). Under rehabilitation purposes, many older adults show higher interests in playing exergames as compared to real-world activities (Wollersheim et al., 2010). The feasibility and concept of VR systems are linked to the simulation, interaction, and immersion of the existing technologies (Soltani, 2019). While earlier exergame systems were using TV screens to project the game in front of the players, newer systems employ portable head-mounted displays (HMD) with larger fields of view (FOV) and stereoscopic visual fields, that are updated continuously using head position and rotation. By adding depth perception and by blocking external visual information, these systems may offer acceptable, feasible, and ecologically valid results in addition to the previous assessment methodologies.

In the early 2000s, Takahashi and Murata (2001) suggested that VR experience using HMD systems may cause symptoms of motion sickness and poor equilibrium. Since HMD systems block the eyesight, the users may lose their balance by hitting physical objects. The immersive virtual environments (VE) may also cause conflicts between proprioceptive and vestibular sensory systems. This can also reduce users' abilities to regain stability after balance loss (Keshavarz et al., 2015). The illusory sensation of body movement induced by virtual scenarios, such as riding a VR rollercoaster, may also cause loss of balance. Participants might try to accommodate these illusory self-movements with physical body movements, which can cause them to lose their balance. Therefore, it is important to understand how the type of display affects motor behavior, especially if the system is being used as training and rehabilitative tools for older adults. In this systematic review, our goal was to investigate the validity, reliability, safety, feasibility, and efficacy of HMD systems to assess and train balance in older adults.

## Methods

This systematic review was conducted according to the Preferred Reporting Items for Systematic Reviews and Meta-Analysis (PRISMA) guidelines (Moher et al., 2009). There is no registered protocol for this review.

### Eligibility Criteria

The eligibility criteria were formulated by using a modified patient population, intervention/indicator, comparator, outcome, and study design (PICOS) framework (Santos et al., 2007). The population comprised older adults of 50+ years of age, and the intervention/indicator was the assessment or training of balance using HMD, which was compared with traditional assessment or other training programs. By using exergames (e.g., Nintendo Wii or Xbox Kinect), participants can still receive contextual information from their surrounding and adjust their balance accordingly. Therefore, articles that used other VR technologies, such as exergames, for presenting the VR scenarios were excluded. The outcomes that we considered were balance control or the ability to maintain balance. We considered full-text articles with different designs that investigated balance assessment or training. While we considered conference papers (if full article was available) for inclusion, those that provided only the abstract (conference abstract) were excluded. We also excluded review articles.

### Search Strategy

The EBSCOhost, Scopus, Web of Science, and PubMed electronic databases were searched for articles and conference abstracts that focused on the use of HMD to assess and train balance and were published between 1990 until 1 September 2020. The following search terms and Boolean operators were used: (HMD OR head mount^*^ display OR virtual reality OR artificial environment OR simulated 3D environment OR simulated three-dimensional environment) AND (balance OR posture OR fall) AND (vestibular OR visual OR somatosensory OR context^*^). Appendix 1 details the search strategy used. The search was restricted to English peer-reviewed articles. Each author independently screened the titles and abstracts of the identified records for relevance. The full text of the articles that appeared relevant and those without obvious relevance (after inspecting the title and abstract) were retrieved. The full-text records were read, and their eligibility was screened with the criteria mentioned above. The reference lists of all eligible studies were hand-searched to identify additional relevant studies. Any disagreement was resolved by mutual discussion.

### Data Extraction

One author (PS) extracted all data from each article into a piloted form. The data extracted comprised the type, number, age, and sex of the participants, HMD type, VR scenario, protocol, main outcomes, and the actual HMD effect. Due to the heterogeneity on the population characteristics, HMD interventions, and outcome assessment tools, a meta-analysis was not pursued.

### Methodological Quality and Risk of Bias

The methodological quality of each article was assessed using a custom quality assessment questionnaire adapted from Downs and Black (1998) and Campos et al. (2011). These questionnaires provide scores for study quality, and internal and external validity, in randomized and non-randomized studies. The results from these questionnaires provide insights on the completeness of the reporting which helps future studies on commonly overlooked methodological steps, and how future studies should comprehensively report their methodology. Each article was evaluated on 13 questions that considered the reporting parameters, internal and external validity, and study power (Table 1). Each question was answered by “Yes,” “No,” or “Unable to determine.” As this review represents a qualitative summary of using HMD in assessing and training balance, no total score was computed for quality assessment. The risk of bias was appraised using the Risk of Bias Assessment tool for Non-randomized Studies (RoBANS; Kim et al., 2013). The RoBANS tool can be applied to different types of studies and eliminates the need to use several tools for each type of study. We opted to use solely the RoBANS tool to display all the risk of bias judgements in a single tool. The RoBANS tool includes the following domains: selection of the participants (selection bias), confounding variables (selection bias), measurement of exposure (performance bias), blinding of outcome assessments (detection bias), incomplete data outcome (attrition bias) and selective outcome reporting (reporting bias). For intervention studies, we also considered two additional domains to account for bias arising from the interventions, which included planning and implementation of interventions (performance bias) and deviation from intended interventions (performance bias). Each domain was judged as unclear, low risk, or high risk of bias. Any disagreements were discussed until consensus.

**Table 1:** Quality assessment questionnaire.

	**Questions**
Q1	Is the hypothesis/objective/aim of the study clearly described?
Q2	Are the main outcomes to be measured clearly described in the Introduction or Methods?
Q3	Are the characteristics of the participants clearly described?
Q4	Are the inclusion/exclusion criteria described and appropriate?
Q5	Are the main findings of the study clearly described?
Q6	Are estimates of the random variability in the data for the main outcomes provided?
Q7	Have actual probability values been reported for the main outcomes?
Q8	Are the participants representative of the entire population from which they were recruited?
Q9	Are the setting and conditions typical for the population represented by the participants?
Q10	Are retrospective unplanned analyses avoided?
Q11	Are the statistical tests used to assess the main outcomes appropriate?
Q12	Are the main outcome measures used accurate (valid and reliable)?
Q13	Is a sample size justification, power description, or variance and effect estimates provided?

## Results

### Search Results

**Figure 1:**
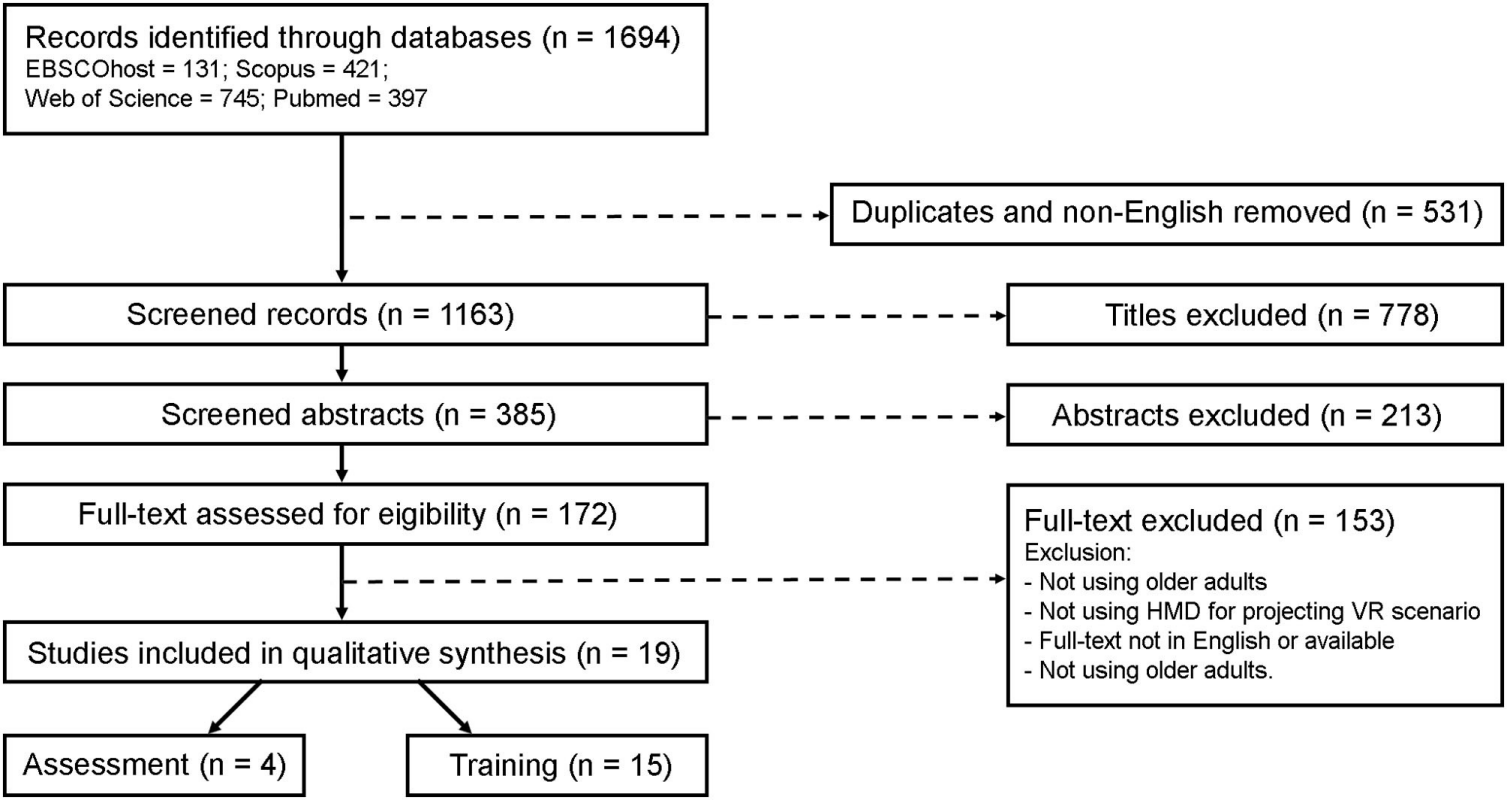
Flowchart of the obtained results, unfolding the search strategy.

A total of 1,694 articles were found in the initial search. After duplicated and non-English removal, 385 articles were screened for titles and abstracts. After full text review, a total of 19 articles evaluating the use of VR HMD in balance assessment and training in older adults were included (Figure 1).

### Methodological Quality and Risk of Bias Assessment

**Figure 2:**
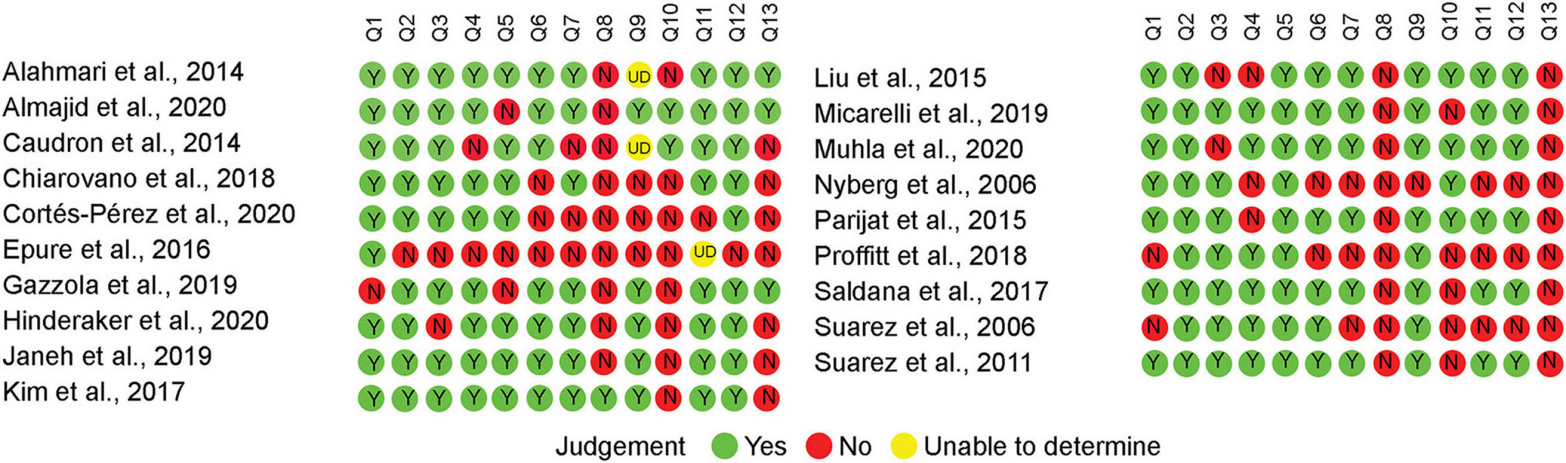
Quality assessment of the included studies.

The results of the quality assessment are mentioned in Figure 2. Most of the studies properly described their aims, main outcomes, participants' characteristics, inclusion/exclusion criteria, and main findings (Q1 to Q5, respectively). For reporting the information (Q6 and Q7), most of the studies mentioned that their data were either normally distributed or provided standard error, standard deviation, confidence interval, and actual probability values. External validity was measured using Q8 and Q9. Since most of the studies were only using older adults without comparing them with a younger control group, the distribution of the main confounding factors was not the same in the study sample. Finally, internal validity was evaluated using Q10 to Q13. Very few studies were based on a predefined hypothesis or provided justification for their sample size, power description, variance, and effect estimates.

**Figure 3:**
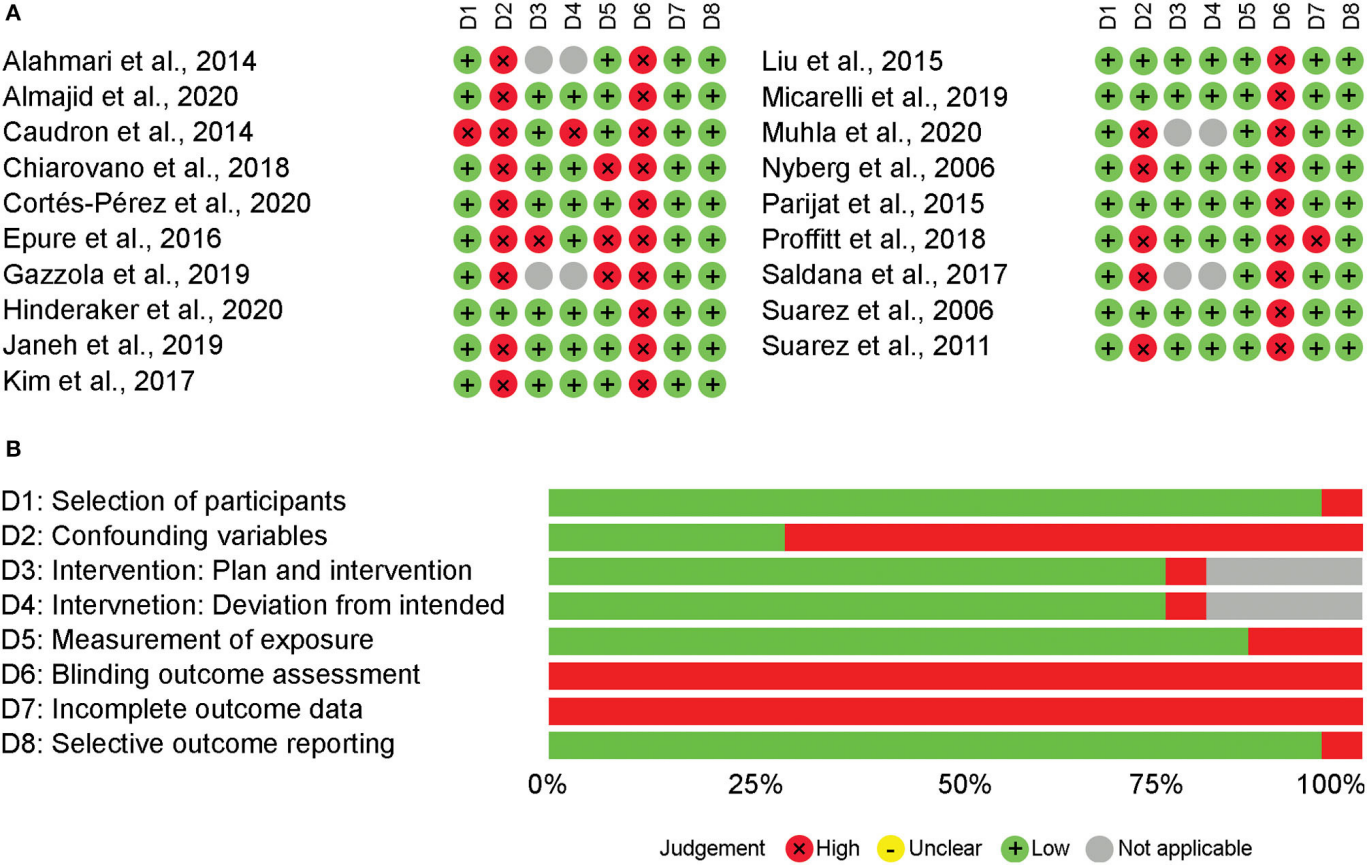
Risk of bias appraisal using RoBANS. Risk of boas domain judgement for each study using RoBANS **(A)**. Summary appraisal of risk of bias domains using RoBANS **(B)**.

The judgement of each domain for each study, as well as the summary of each domain can be seen in Figure 3. Note that in the summary of domain risk of bias there is a high percentage (~20%) of “not applicable,” which is related to the non-intervention studies; this high percentage is due to the higher sample size of these studies that translated into greater weight in the summary plot. All studies showed high risk of detection bias because none of the studies blinded the outcome assessor. While selection bias due to uncontrolled confounding variables was a major concern (74% of studies), selection bias due to selection of participants had low risk of bias all but one study. Attrition and reporting bias were also not common, with only one study judged as high risk of bias attrition bias and none for selective reporting. Performance bias due to measurement of exposure was judged as high risk in 16% of the studies. From the 15 studies that included HMD as an intervention, only two studies were judged to having high risk of performance bias due to planning and implementation of interventions or deviations from intended interventions.

### Participants Characteristics

The included studies presented a wide variety of health conditions and age ranges (Table 2). Eleven studies involved healthy older adults (58%; *n* = 230), four studies included individuals with vestibular disorder (21%; *n* = 193), four studies included individuals with Parkinson's disease (PD; 21%; *n* = 67), one study included participants with mild cognitive impairment (MCI; 5%; *n* = 12), two studies involved participants with stroke (10%; *n* = 8), and five studies included balance impaired and at risk of fall individuals (26%; *n* = 127).

**Table 2:** List of the studies with the data extracted from each article.

**References**	**Health status or pathological condition**	**Number**	**Age**	**Sex**	**HMD sensor**	**VR scenario/Avatar**	**Protocol**
Alahmari et al. (2014)	Healthy	30	77.2 ± 5.0	18F, 12M	BRU, N/R	Stationary visual scene (basketball gym), No avatar	CoP area and velocity, comparison with SOT
Almajid et al. (2020)	Healthy	16	69.0 ± 4.4	8F, 8M	Oculus Rift DK 2	Visual disturbance consisting of falling snowflakes, No avatar	BBS, TUG test with additional motor task (holding a cup)
Gazzola et al. (2019)	Healthy	41	72.51 ± 6.84	24F, 17M	BRU, eMagin Z800 3D Visior	Following random visual targets with different colors and letters, No avatar	CoP, LoS, VoS
Hinderaker et al. (2020)	Healthy	10	71.0 ± 5.0	6F, 4M	HTC Vive	Moving colored spherical particles, No avatar	CoP
Kim et al. (2017)	Healthy	11	66.0 ± 3.0	8F, 3M	Oculus Rift DK2	Walking in VE, No avatar	Mini-BESTest, CoP, SSQ, stress, and arousal
Liu et al. (2015)	Healthy	36	71.24 ± 6.82, 70.54 ± 6.63, 74.18 ± 5.82	18F, 18M	Sony Glasstron	VE with visual perturbations, No avatar	CoM velocity
Muhla et al. (2020)	Healthy	31	73.7 ± 9.0	20F, 11M	HTC Vive	Sit and get up task, No avatar	TUG
Nyberg et al. (2006)	Healthy	4	69.0-80.0	2F, 2M	Virtual Research VR8	Walking in VE, heavy snow vs. tilting world, graphic disturbance and flickering, No avatar	Disturbances of balance, walking patterns
Parijat et al. (2015)	Healthy	24	65.0+	12F, 12M	Sony Glasstron	Exploration scene with visual perturbation, No avatar	Gait
Saldana et al. (2017)	Healthy	8	81.4 ± 6.25	7F, 1M	Oculus Rift DK2	Standing activities with semi realistic graphics, No avatar	SSQ, postural sway
Suarez et al. (2011)	Healthy	19	62.3 ± 12.7	N/R	BRU, N/R	Static visual field with visual optokinetic stimulation, No avatar	LoS, CoP, BFR
Alahmari et al. (2014)	Vestibular	15	66.0 ± 8.0	10F, 5M	BRU, N/R	Stationary visual scene (basketball gym), No avatar	CoP area and velocity, comparison with SOT
Caudron et al. (2014)	Idiopathic PD	17	61.9 ± 8.2	7F, 10M	Solo, Myvu	Simplified avatar of participant's body using motion capture, Simplified avatar	Pull task with eyes open, eyes closed, or visual feedback
Chiarovano et al. (2018)	Vestibular impairment (neuritis), cervicogenic dizziness, general dizziness and imbalance	90	65.0 ± 15.0	51F, 39M	Samsung Gear VR	Stereoscopic visual scene, No avatar	Several visual conditions and unpredicted visual perturbation at several amplitudes of movement
Cortés-Pérez et al. (2020)	Acute stroke	3	45, 50, 53	3M	HTC Vive	Interaction with virtual objects while standing, With animal avatar	BBS and Tinetti scale, SVV, Romberg, gait, ABC, FES-I, and perception of verticality
Epure et al. (2016)	Balance impaired	6	59.0-69.0	N/R	N/R	Skiing game, semi realistic graphics, No avatar	Physical balance
Gazzola et al. (2019)	Chronic vestibular disorder	76	71.90 ± 5.23 and 73.92 ± 6.27	55F, 21M	BRU, eMagin Z800 3D Visior	Following random visual targets with different colors and letters, No avatar	CoP, LoS, VoS
Janeh et al. (2019)	PD	15	67.6 ± 7.0	15M	HTC Vive	Walking in VE, No avatar	SSQ, gait
Kim et al. (2017)	PD	11	65.0 ± 7.0	8F, 3M	Oculus Rift DK2	Walking in VE, No avatar	Mini-BESTest, CoP, SSQ, stress, and arousal
Micarelli et al. (2019)	UVH and MCI	12, 12	74.3 ± 4.7 and 72.5 ± 3.6	6F, 6M and 7F, 5M	Revelation 3D	Track speed racing 3D game, 110° FOV, No avatar	VOR, postural control
Proffitt et al. (2018)	Post-stroke patients	5	56.0 ± 3.0	2F, 3M	Oculus Rift DK2	Recycling game (sorting, filling, and loading), No avatar	Interview, usability
Saldana et al. (2017)	At risk of fall	5	78.4 ± 9.37	3F, 2M	Oculus Rift DK2	Standing activities with semi realistic graphics, No avatar	SSQ, postural sway
Suarez et al. (2011)	Balance disorder	26	73.0-82.0	N/R	BRU, N/R	Virtual scene under different sensory conditions, No avatar	CE, SV
Suarez et al. (2011)	PD	24	66.5 ± 8.5	N/R	BRU, N/R	Static visual field with visual optokinetic stimulation, No avatar	LoS, CoP, BFR

*ABC, Activities-specific balance confidence; BBS, Berg balance scale; BFR, Balance functional reserve; BRU, Balance rehabilitation unit; CE, Confidential ellipse; CoB, Center of balance; CoP, Center of pressure; EMG, Electromyography; FES-I, Falls efficacy scale international; FOV, Field of view; fps, Frames per second; HMD, Head-mounted display; LoS, Limit of stability; MCI, Mild cognitive impairment; Mini-BESTest, Mini-balance evaluations systems test; MSSQ, Motion sickness susceptibility questionnaire; N/R, Not reported; PD, Parkinson's disease; SOT, Smart equitest sensory organization test; SSQ, Simulator sickness questionnaire; SV, Sway velocity; SVV, Subjective visual vertical test; TUG, Timed up and go test; UVH, Unilateral vestibular hypofunction; VE, Virtual environment; VOR, Vestibul-ocular reflex; VoS, Velocity of oscillation; VR, Virtual reality*.

### HMD and VR Protocols

Most of the included studies used the Oculus Rift (DK 1 and 2) as HMD tool (33%). Other devices included HTC Vive (19%), Balance Rehabilitation Unit (BRU; 33%), and other systems (eMagin Z800 3D Visior, Sony Glasstron, Virtual Research VR8, Solo Myvu, Samsung Gear VR, and Revelation 3D; 43%). Twenty-nine percent of the studies did not report the name of the HMD. The VR scenarios were heterogenous across studies. Seven studies (37%) used VEs with adjustable visual perturbances while four studies (21%) used stationary visual scenes. Two studies (11%) used simple avatar VE and one study (5%) used (semi)realistic VEs. In 17 studies (89%), participants had to interact with VR to complete the tasks.

### Outcome Assessment

Sixteen studies (84%) used validated physical test batteries that utilized force plates, plantar pressure, posturography, physiological profile assessment, and human machine interaction. Two studies (11%) used specific perturbation-based protocols either by pulling and pushing, or by including visual perturbations inside the VR scenario. Six studies (32%) used questionnaires including Motion Sickness Susceptibility Questionnaire (MSSQ), Simulator Sickness Questionnaire (SSQ), Falls Efficacy Scale International (FES-I), and Activity-specific Balance Confidence (ABC). Four studies (19%) used surveys including gaming habits, Dynamic Gait Index, Berg Balance Scale (BBS), Tinetti Scale, Subjective Visual Vertical (SVV) test, Romberg, and Motion Sickness Rating. Actual HMD effect varied between studies. Studies that included healthy older adults, measured changes in gait patterns, postural stability, balance, fear of falling, body sway, simulator sickness, and balance strategies. Studies comprising patients with pathological conditions, evaluated both improvement and deterioration of balance and its related metrics.

### Can VR Systems Using HMD Be Used for Assessing Balance?

The studies were heterogenous in terms of HMD features, VR scenarios, and comparison outcomes. Therefore, a direct comparison was not possible. Below, we report the results of HMD systems to assess balance in regard to validity, reliability, and consistency of their measurements with gold standard methodologies (Tables 3, 4).

**Table 3:** Outcomes of the studies and the actual HMD effect in healthy individuals.

**References**	**Balance assessment/training protocol**	**Outcomes**	**Actual HMD effect**
Alahmari et al. (2014)	CoP area and velocity, comparison with SOT	Significant correlation between BRU and SOT (CoP area ICC = 0.64–0.81, velocity ICC = 0.44–0.76), higher CoP area, and velocity for older adults compared to younger adults.	Correlation with traditional test. Need to define new norms for different participants.
Almajid et al. (2020)	BBS, TUG	Non-statistically significant differences between younger and older adults in balance measures and cognitive function, lower BBS in older adults, visually independent older adults perform better that dependent older adults, Lower turning cadence, walking slower, decreased pitch, yaw, and roll peak trunk velocity in all TUG components. Smaller AP and ML acceleration ranges in sit-to-stand and smaller AP acceleration in stand-to-sit.	Wearing HMD negatively affects TUG.
Gazzola et al. (2019)	CoP, LoS, VoS	Higher LoS and lower CoP in control group compared to training groups.	Wearing HMD did not affect balance negatively.
Hinderaker et al. (2020)	CoP	Older adults had maximum amplitude compared to younger adults at the OF speed of 10 m/s, age could affect older adults' abilities to process OF stimulation.	Wearing HMD increased body sway.
Kim et al. (2017)	Force plate, SSQ	Decreased stress after training, lower arousal compared to PD patients (arousal absolute changes in healthy young vs. healthy old = 3 ± 2 vs. 4 ± 4).	Wearing HMD decreased stress, did not change simulator sickness, and static and dynamic balance.
Liu et al. (2015)	CoM velocity	Higher fall reduction and better forward trunk rotations reduction in VR group, better corrections to a slip in traditional methods, lack of efficacy as patients gain experience	Initial positive results but lower efficacy as they gain experience.
Muhla et al. (2020)	TUG	Higher completion times and steps in TUG VR.	Wearing HMD increased completion times and induced more cautious behavior.
Nyberg et al. (2006)	Kinematics, Survey, SSQ	Tilting virtual scene affected balance performance during walking (% increased time to complete task vs. no HMD = 166–283%).	Changes in balance with visual perturbations.
Parijat et al. (2015)	Gait	Increased ankle plantarflexion, knee flexion, and trunk flexion at heel contact in VR compared to overground walking. Early muscle activation was observed in VR compared to treadmill walking.	Wearing HMD induced balance strategies and cautious behavior.
Saldana et al. (2017)	Force plate, SSQ	Lower change of tilt in anteroposterior direction compared to patients with risk of fall (tilt at risk vs. healthy = 0.7°/s vs. 0.4°/s).	Similarity with traditional test. Need to define new norms.
Suarez et al. (2011)	LoS, CoP, BFR	Healthy had lower CoP values compared to the PD group in the static visual field, BFR was reduced significantly in sensory VR scenarios.	Wearing HMD increased risk of falls when viewing sensory VR scenarios.

*ABC, Activities-specific balance confidence; AP, Anteroposterior; BBS, Berg balance scale; BFR, Balance functional reserve; BRU, Balance rehabilitation unit; CE, Confidential ellipse; CoP, Center of pressure; DGI, Dynamic gait index; DHI, Dizziness handicap inventory; FES-I, Falls efficacy scale international; FOV, Field of view; HMD, Head-mounted display; ICC, Intraclass correlation coefficient; ML, Mediolateral; MS, Multiple sclerosis; MSSQ, Motion sickness susceptibility questionnaire; OF, Optical flow; PD, Parkinson's disease; PPA, Physiological profile assessment; SOT, Smart equitest sensory organization test; SPT, Static posturography testing; SRF, Static rest frame; SSQ, Simulator sickness questionnaire; SUS, System usability scale; SV, Sway velocity; SVV, Subjective visual vertical test; VR, Virtual reality; TUG, Timed up and go test*.

**Table 4:** Outcomes of the studies and the actual HMD effect in individuals with pathological conditions.

**References**	**Pathological condition**	**Balance assessment/training protocol**	**Outcomes**	**Actual HMD effect**
Alahmari et al. (2014)	Vestibular	CoP area and velocity, comparison with SOT	Significant correlation between BRU and SOT (CoP area ICC = 0.64−0.81, velocity ICC = 0.44–0.76), higher CoP area, and velocity for younger vestibular adults compared to younger healthy adults.	Correlation with traditional test. Need to define new norms for different participant.
Caudron et al. (2014)	Idiopathic PD	Angular displacement measurement using kinematics (postural reaction peak, final orientation, stability of performance)	No changes in backward trunk tilt (mean amplitude = 9–11°, patients could recover their initial vertical orientation and had less frequent falls with visual feedback.	Less frequent falls immediately after HMD protocol.
Chiarovano et al. (2018)	Vestibular impairment (neuritis), cervicogenic dizziness, general dizziness and imbalance	DHI, postural sway	No correlation between DIH score and balance measurement, significant correlation between DIH and age, and balance and age.	Wearing HMD provided more possibilities for controlled visual conditions.
Cortés-Pérez et al. (2020)	Acute stroke	BBS, Tinetti scale, SVV, TUG, ABC	Higher functional balance (BBS and Tinetti scale) in VR compared to traditional and no treatment, larger reduction of risk of fall in VR patient.	Wearing HMD improved postural balance and gait and induces cautious behavior.
Epure et al. (2016)	Balance impaired	Survey, Kinect and Wii Balance Board	Higher stability when using monitor display compared to HMD (−20–30° vs. −20–40°).	Wearing HMD increased instability.
Gazzola et al. (2019)	Chronic vestibular disorder	CoP, LoS, VoS	Higher LoS in control group compared to training groups, higher CoP area and VoS in vestibular patients with a history of fall	Wearing HMD did not affect postural ability of vestibular patients without falls negatively, worsens postural ability of vestibular patients with falls, could be used to identify and quantify patients at risk of falling.
Janeh et al. (2019)	PD	SSQ, gait	Higher step width, cadence, gait variability, and gait pattern insecurity for PD patients in VR. Low SSQ and no significant increase over the time of experiment.	Wearing HMD increased gait symmetry with the help of visual scenes.
Kim et al. (2017)	PD	Force plate, SSQ	Decreased stress and higher arousal for PD patients (stress levels pre- and post = −9 ± 4 vs. −11 ± 3; arousal absolute changes in PD patients = 8 ± 7).	Wearing HMD decreased stress, did not change simulator sickness, and static and dynamic balance.
Micarelli et al. (2019)	UVH and MCI patients	SPT, DHI, ABC, DGI, SSQ	Improvement in otoneurological outcome measures, no changes in VOR gain.	Improved balance compared to traditional training.
Proffitt et al. (2018)	Post-stroke patients	Survey	Visual perception played a role in patients' preference, no adverse effects in balance.	Wearing HMD did not cause adverse effects, post-stroke patients need assistance.
Saldana et al. (2017)	At risk of fall older adults	Force plate, SSQ	Higher change of tilt in anteroposterior direction in patients with risk of fall (tilt at risk vs. healthy = 0.7°/s vs. 0.4°/s).	Similarity with traditional test. Need to define new norms.
Suarez et al. (2011)	Balance disorder	CE, SV	Higher reductions in postural responses after visual optokinetic stimulation	Wearing HMD resulted in improvements in postural control.
Suarez et al. (2011)	PD patients	LoS, CoP, BFR	PD had higher CoP values compared to the control group in the static visual field, BFR was reduced significantly in sensory VR scenarios.	Wearing HMD increased risk of falls when viewing sensory VR scenarios.

*ABC, Activities-specific balance confidence; BBS, Berg balance scale; BFR, Balance functional reserve; BRU, Balance rehabilitation unit; CE, Confidential ellipse; CoP, Center of pressure; DGI, Dynamic gait index; DHI, Dizziness handicap inventory; FES-I, Falls efficacy scale international; FOV, Field of view; HMD, Head-mounted display; ICC, Intraclass correlation coefficient; MS, Multiple sclerosis; MSSQ, Motion sickness susceptibility questionnaire; PD, Parkinson's disease; PPA, Physiological profile assessment; SOT, Smart equitest sensory organization test; SPT, Static posturography testing; SRF, Static rest frame; SSQ, Simulator sickness questionnaire; SUS, System usability scale; SV, Sway velocity; SVV, Subjective visual vertical test; TUG, Timed up and go test; VoR, Vestibulo-ocular reflex; VoS, Velocity of oscillation; VR, Virtual reality*.

#### Validity and Reliability of HMD Systems for Assessing Balance

Only one study evaluated the validity and reliability of HMD systems for assessing balance. Alahmari et al. (2014) measured test-retest reliability, concurrent validity, and construct validity of their HMD by comparing it to the center of pressure (CoP) measurement in healthy adults and individuals with vestibular disorders. The CoP area and velocity in anteroposterior and mediolateral directions were correlated with the measurements of SOT, indicating concurrent validity with a standard clinical test for measuring sensory organization abilities in healthy and vestibular patients.

#### Validity and Performance of HMD Comparing to Traditional Balance Tests

Two studies compared the validity and performance of HMD to a traditional balance test—the Timed Up and Go (TUG) test. These two studies showed that adding the HMD VR component to the TUG, the task was more challenging to complete and that the self-motion perceptions during the task changed. Muhla et al. (2020) contextualized the traditional TUG to make it closer to the real-world scenarios. They observed an increase in the number of steps and the time to complete the VR TUG test. Almajid et al. (2020) added a motor task and visual stimulus to the TUG test and evaluated the effects of age-related visual dependence on motor performance. They showed that while wearing HMD, older adults' egocentric self-motion perceptions decreases which could negatively affect motor performance. Wearing HMD also changed biomechanical and perceptual constraints, limited peripheral vision, and created inaccurate subject-to-object and object-to-object distance estimation.

#### Capacity of HMD Systems in Differentiating Healthy and Balance-Impaired Individuals

Only one study evaluated the capacity of HMD systems to differentiate between healthy individuals and those with balance impairments. To this end, Saldana et al. (2017) assessed the validity and reliability of their VR HMD system by comparing the balance measurements with a force-plate. They showed that patients with risk of falls display faster anteroposterior velocity as compared to the healthy individuals. This finding was associated with increased odds of falling and thus capable of identifying those with increased risk of falls.

#### Effects of VR Elements and Scenarios

The effects of VR elements and scenarios was investigated in two studies comprising either healthy or balance-impaired individuals. Complexity of VE can also increase the time to complete the tasks in older adults (Nyberg et al., 2006). Similarly, walking patterns including walking speed and stride length, balance reactions, and slips could vary according to the equipment, sensory load, and the VR scenarios (Nyberg et al., 2006). Gazzola et al. (2019) measured the effects of visual, somatosensorial, and visual-vestibular manipulation on postural control in older adults. Vestibular patients with(out) history of falls had lower limit of stability, which is the area where their oscillation is safer. They showed that visual and somatosensory cues could compensate the inaccurate information of the vestibular system for the maintenance of body balance.

#### Usability of HMD Systems

Only two studies assessed the usability of HMD systems to measure balance. Saldana et al. (2017) measured the acceptability of their VR system using a simulator SSQ. They reported no significant changes in the nausea-subscale score and SSQ overall score after VR exposure. Kim et al. (2017) used longer bouts of walking in VR and showed that symptoms of simulator sickness were higher in patients with PD compared to healthy younger and older adults.

### Can VR Systems Using HMD Be Used as a Balance Training Tool?

#### Safety and Feasibility

Several studies investigated the safety of HMD systems for training balance. Kim et al. (2017) showed that within their setup with simple cityscape and without any turns, doorways, or crossing thresholds, most of the patients with PD completed the tests without any discomfort. Proffitt et al. (2018) used their HMD system for telerehabilitation of a group of post-stroke patients. They showed that all patients required assistance for balance and fall prevention that limit the application of HMD systems as telerehabilitation interventions. The duration of VR exposure was an important factor that could affect the feasibility of balance training using HMD systems. Other studies have analyzed the balance performance of individuals with balance-related pathological conditions and explored which strategies can be employed to complete the VR tasks. Nyberg et al. (2006) studied fall tendencies of healthy individuals in functional VR settings, such as walks and turns, and showed that their subjects usually opted for more cautious strategies to prevent a fall incident.

#### Effects of the HMD Display Features and VR Scenarios

The HMD features have been shown to significantly affect the balance training outcomes. Epure et al. (2016) compared the effects of display type on physical balance in healthy and balance-impaired adults. They showed that both groups had higher stability when using monitor displays compared to HMD. Different VR scenarios yielded different outcomes. Janeh et al. (2019) used VR-based gait manipulation to improve gait symmetry in a group of patients with PD. Their VR task dissociated visual and proprioceptive inputs and increased the patients' step length, cadence, and swing time variabilities for both body sides. VR gaming systems with visual modifications also improved vestibulo-ocular reflex and postural control in individuals with vestibular disorders or mild cognitive impairments (Micarelli et al., 2019).

The way that the VR scenario is perceived (first vs. third-person view) can have an impact on the balance outcome, but the results were individual-dependent. While some post-stroke patients felt more engaged when playing in a first-person view, others felt more in control and at ease when playing in the third-person view (Proffitt et al., 2018). Changes of visual perception was found to be an important feature of HMDs and VR scenarios. Suarez et al. (2011) evaluated the effects of stable and moving visual fields on balance outcomes of patients with PD. They showed that changes in visual information increased the CoP area which triggered balance control. In another study, Suárez et al. (2006) evaluated postural adaptations after vestibular rehabilitation in two different perceptual conditions with static and dynamic visual fields, and found that visual fields with moving targets could elicit postural disturbances. Parijat et al. (2015) induced virtual perturbations similar to slip and observed evoked recovery reactions that could be transferred to the actual slip trials. Hinderaker et al. (2020) evaluated the effects of optical flow speed on brain activity and postural control of younger and older adults. They showed that older adults show higher brain activities at lower optical flow speeds as compared to younger adults, but that the optical flow speed did not affect the postural sway in either the younger or older adults.

#### Effectiveness of HMD Balance Training vs. Other Current Methods and Training Systems

A total of six studies compared the effectiveness of VR HMD systems with traditional training protocols (Tables 3, 4). In these studies, HMD had good or better outcomes compared to the traditional training programs. In a small cohort, Cortés-Pérez et al. (2020) compared the effects of VR balance training with conventional physiotherapy in three stroke patients. After 2 months, the patient under VR training showed higher improvements of balance and obtained higher walking speed as compared to those with conventional physiotherapy. Chiarovano et al. (2018) in a cohort of balance-impaired individuals, evaluated the relationship between objective and subjective measurements of balance while perturbed visual inputs were introduced using VR. They found no correlations between objective and subjective balance measurements but showed that VR allowed revealing the participants with the real risk of fall. Their system categorized patients in three groups: those with high subjective measurements who passed the objective test, those with low subjective measurements who failed the objective test, and those with a correlation between subjective and objective measurements. Caudron et al. (2014) evaluated whether postural responses of patients with PD could be improved by traditional focus-based instructions or visual biofeedback. Biofeedback visual training improved stabilization and orientation components of postural control as compared to the traditional method of focus-based instructions. They also observed that online visualization of trunk and head orientations improved the stabilization when postural disturbance occurs. Liu et al. (2015) compared the effects of two slip training modalities on reducing fall frequency and reactive recovery. While both moving platform and VR training groups reduced their fall frequencies after training, VR group also reduced their forward trunk rotations which has been shown to bring the center of mass (CoM) of the body within boundaries of stability. Micarelli et al. (2019) compared traditional vestibular rehabilitation to VR balance training. They showed that by modifying visual information and increasing the complexity of their VR protocol, regardless of their health status, they could achieve significantly higher VOR gains as compared to those undergoing traditional vestibular rehabilitation alone.

## Discussion

### Summary

The purpose of this systematic review was to explore the validity, reliability, safety, feasibility, and efficacy of HMD systems for assessing and training balance in older adults. The combined findings of the included studies show that VR HMD systems offer ecologically valid scenarios to assess and train functional balance. There is however a need to define standardized norms and protocols according to age, health status, and severity of disease. The studies also showed that several parameters of display type, VR scenario, and the duration of exposure can contribute to the safety and feasibility of HMD VR systems for balance training. These features could be adjusted according to participants' needs to ensure safety and efficacy of training. The use of HMD systems was effective in training balance and can be a useful tool to augment previously established interventions. Various visual scenarios can be added, removed, isolated, and manipulated to identify and treat specific balance-related impairments of different clinical conditions. The level of difficulty can also be adjusted to the patients' baseline levels to allow progress or regress when needed.

### Validity and Effectiveness of HMD VR Systems for Measuring and Training Balance in Healthy Older Adults

Only one study showed that HMD VR systems were valid and effective in healthy older adults. Those who trained with HMD VR systems showed improved gait parameters and lowered frequency of falls (Giotakos et al., 2007; Parijat et al., 2015; Kim et al., 2017). These improvements are probably due to the challenges that are induced when using VR scenarios. While some researchers reported that older adults have similar balance performance in traditional and VR scenarios (Alahmari et al., 2014), others have argued that participants' behaviors in VR versions of balance tests differ significantly (Muhla et al., 2020). One explanation could be that HMD systems remove the surrounding visual cues that older adults require for maintaining balance. Older adults will have to rely on VR scenarios, presented through HMD systems, which offer different visual stimulation compared to the real world. Another explanation is that HMD VR systems evoke various proactive and reactive postural adjustments (Liu et al., 2015; Parijat et al., 2015). It has been shown that participants generally walk slower, and their performance is lower in VR balance tests as compared to the traditional ones (Nyberg et al., 2006; Almajid et al., 2020). Participants also acquire more cautious behaviors when completing VR tasks in less secure VEs (Muhla et al., 2020) and take more time and steps which are adaptive strategies to ensure balance and avoid falling (Nyberg et al., 2006). Other strategies that healthy adults employed for increasing the stability in unfamiliar situations included a reduced forward displacement of whole-body CoM and lowered height of CoM (Thomas et al., 2016).

Motion sickness is believed to happen when the brain's assumption about sensory information does not match the actual received signals (Reason, 1978). This mismatch could be due to hardware and software limitations of VR HMD systems. The motion-to-photon latency, or the delay between the users' movements and their reflections on the display, can contribute to the motion sickness (Choi et al., 2018). The effects of latency are so important that even the smallest lag degrades the sense of balance compared to the naked eye with a similar FOV (Kawamura and Kijima, 2016). The interactions of gender and sickness history can also influence the risk of motion sickness and instability and should be considered when designing the HMD hardware and software (Kawamura and Kijima, 2016; Munafo et al., 2017). It has been shown that females are more susceptible to motion sickness (Munafo et al., 2017). In pathological conditions, such as PD, the sensory deficits could make the individuals less prone to the simulator sickness and could be less influential on their perceptions of balance (Kim et al., 2017). On the other hand, HMD related head movements pose sensory mismatch to the central nervous system of UVH patients, which later tries to resolve the mismatch (Clendaniel, 2010).

The duration of VR exposure should also be carefully monitored as it can provoke sickness symptoms. Previous research has associated verbal reports of cybersickness severity, as well as relatively high incidences of simulator sickness with the duration of HMD use (Treleaven et al., 2015; Dennison et al., 2016). HMD systems could also cause visual fatigue due to a disruption of the natural relationship between vergence and accommodation (Mon-Williams and Wann, 1998). Various physiological measurements have been used to estimate post-immersion cybersickness (Dennison et al., 2016). Those with greater levels of cybersickness may show less variations in postural sway (Dennison and D'Zmura, 2017). Therefore, the physiological measurements are important for differentiating between motion sickness and actual imbalance (Dennison et al., 2016).

While postural instability increases with time, it only happens when visual perturbations are present. It seems that elements of VR scenarios could also affect the way healthy older adults interact with the systems. Some researchers reported that increasing rotation speed in VR scenarios could increase cybersickness (Dennison and D'Zmura, 2017). Others have shown that speed manipulations in simple and complex VR scenarios do not cause motion sickness (De Keersmaecker et al., 2020). Adding an independent visual background to the scene could also reduce postural disturbance and could reduce the simulator sickness (Prothero et al., 1999; Duh, 2001).

As compared to younger adults, the older population is relatively more egocentric and more accurate when they are in the first-person perspective compared to the third-person perspective (Mattan et al., 2017). Research has also highlighted that a sense of actual presence in VR could be weakened if older participants view the VE from a third-person perspective (Lenggenhager et al., 2007). Therefore, perspective-taking capacity should be considered when designing virtual scenarios for older adults. Concerning the interaction with avatars, only one study included animal avatars (Cortés-Pérez et al., 2020). It seems that other contextual information around the avatars, including ground and objects, could act as visual reference points for the participants to adjust their balance. Naturalness of the movements, or the alignment between visual and proprioceptive senses, is also reduced when using HMD systems (Sander et al., 2006). Mismatch in proprioceptive and visual senses, or the inability to get information from body, as well as the low FOV and stability of the headset, could also reduce the naturalness of the movements. Additional motion tracking sensors, such as Leap Motion, can embed hands' motions directly into HMD systems, which can act as visual reference points for older adults (Scheggi et al., 2015). After a few trials, participants start to adapt to the gaming and protocol mechanics and adjust their walking to the VR and visual perturbations (Liu et al., 2015). As a result, VR might lose its ability to induce perturbations after 2–3 trials. It is thus important that HMD VR trainings use different scenarios and intensities so that they reduce the risk of adaptation to the VR environments and allow progression to optimize the results.

### Validity and Effectiveness of HMD VR Systems to Measure and Train Balance in Pathological Conditions

There is no standard protocol for defining postural instability in patients with different pathologies. Therefore, the results of different studies are conflicting regarding both assessment and training paradigms.

Fear of falling and perceived postural threat can affect gait patterns in older adults and could induce stiffing strategies in their joints. VR exposure therapy scenarios could increase self-efficacy beliefs of falling and provide a sense of control over falling (Giotakos et al., 2007). In the case of PD and in addition to imbalance issues, many patients have lower access to visual cues. They might have a fear of imbalance and decreased gait performance, preventing them from benefiting from rehabilitation. Patients with neurological conditions (PD and stroke) show improvement of balance and reduction of the risk of falls after VR training as compared to non-VR training (Caudron et al., 2014, Cortés-Pérez et al., 2020). Vestibular patients who are suffering from chronic balance impairments are likely to benefit from HMD VR training. Those who trained with HMD showed overall improvements in vestibulo-ocular reflex gain and in posturography parameters (Micarelli et al., 2017; Viziano et al., 2019). Vestibular patients without MCI have also shown improvements in otoneurological outcome measures after HMD VR training (Micarelli et al., 2019).

The HMD VR scenarios could also help in identifying different patients within certain pathology. For example, Chiarovano et al. (2018) could identify three types of vestibular patients: those with high dizziness scores who did not fail the balance test, those with low dizziness scores who failed the test, and other participants whom their dizziness scores were correlated with balance measurements. Visually dependent older adults with greater risks of fall show smaller acceleration ranges in VR TUG test compared to visually dependent older adults without any risks (Almajid et al., 2020). Patients with PD also show different CoP and balance functional reserve values in static and dynamic VR scenes (Suarez et al., 2011). The differences in the balance-related measures could be used to identify patients with higher balance impairments, to understand who will benefit the most from HMD VR training and guide users on how to adjust the training scenarios to the patients' specific needs. Nevertheless, there is a need for further studies to establish realiable cut-offs for each of these balance-related metrics and to adjust them to the associated pathological disorders. Then, the benefit of using VR is that sensory loads can be tailored and increased according to individuals' visual perception and threshold values to enhance balance performance (Proffitt et al., 2018). Notwithstanding the results of these studies showing the usability of HMDs to identify balance-impaired individuals, there are also conflicting results. Some researchers have shown that the CoP area and velocity were similar to healthy older adults during traditional and VR tests (Alahmari et al., 2014) and others have shown that CoP values were worse in vestibular patients compared to healthy older adults (Gazzola et al., 2019).

As patients get accustomed to the training, postural responses tend to adapt to the visual scenarios (Suárez et al., 2006). It has been suggested that different VR HMD systems could be more salient by improving stimulus presentation through encouraging participants to explore the VE (Menzies et al., 2016). Instructing participants to follow targets that move out of their direct FOV could also increase their presence. VR tasks should also challenge known sensorimotor deficits of individuals with special clinical and functional needs. For example, in patients with PD, more challenging walking tasks like turning, obstacle handling, or passing through doorways are impaired (Mirelman et al., 2011). Similar VR scenarios should be incorporated in the rehabilitation programs to increase their clinical relevance for their targeted population. Different types of virtual scenarios and frequencies of movement disturbance could also affect participants' stability (Jurkojc et al., 2017). It should be noted that participants get accustomed to these disturbances faster in open (seeing the horizon in 100 m distance) compared to closed sceneries (a room; Jurkojc et al., 2017). It is therefore important that patients are exposed to various and progressive training scenarios. The VR tests are easier to standardize than ordinary tests performed in gymnasiums or rehabilitation training facilities, which is a great advantage. Using VR systems, lighting conditions, room size, and other features can be set according to desired specifications. Each condition can also be manipulated individually to calculate their contribution in balance outcomes (Ferdous et al., 2018) and help to further understanding of complex mechanisms involved in balance.

### Challenges and Practical Implications

Older adults may suffer from technological illiteracy. The reluctance of not using VR, stems from common beliefs about required equipment, financial costs, and unfamiliarity with the benefits of VR systems (Schwartzman et al., 2012). An insufficient knowledge of the systems both by the patients and their therapists could also hamper their acceptance. Therefore, VR training systems should have user-centric and user-friendly design. Developers should focus on improving the usability and accessibility of VR training systems, for promoting adherence and motivation. As participants' behaviors in VR differ from traditional environments, new norms should be defined to compare different participants. Different amounts of time spent in the VR rehabilitation programs, as well as motivation and other psychological parameters may also affect the outcomes and practitioners should consider them when interpreting the results.

Researchers, developers, and practitioners should also consider participants' gender and age, as well as sickness and medication history because one system and scenario may not work for everyone. Carefully planned visual scenarios with right frequencies (speed) and duration should be used to prevent visual fatigue and cybersickness. The timing and intensity of introducing new VR elements and scenarios should also be considered when developing training programs to ensure a rate of adaptation that allows continued progression and maximizes training efficacy. Future VR systems should be able to adjust these scenarios and their complexity, as well as sensory load, duration, and game algorithm for different participants. As technology is advancing rapidly, future HMD and processing systems will be faster, smaller, more powerful, and less obtrusive. Hopefully lower latency could be translated in more realistic interactions with VR HMD systems.

### Recommendations for Future Research

Almost all the studies display statistical limitations that limit external validity of their findings. Future studies should include more subjects, control confounding variables, and match for sex to enhance the generalizability of their findings. The effects of other features of VR, such as field of regard, display size, head-based rendering, stereoscopy, realism of lighting, and scene update latency, on balance should be further explored. Future research should also target the effects of biofeedback features, including audio and vibrotactile biofeedback, on postural control. It is also important to explore whether participating in VR trainings translates into functional improvements in real life. Despite the increasing amount of studies in this area, there is still much room to improve the current technologies, and the limitations identified in this review, should be considered when planning future studies.

### Study Limitations

There are a few study limitations to note. The low samples sizes and the high risk of confounding from most studies are the major concerns, and limit both the internal and external validity of findings. The aims and populations of the included studies were heterogenous, which hinder generalizing the results. Many clinical and balance-related measurements could be influenced by medication and severity of the disease. Inevitably, healthy and unhealthy older adults might have different SSQ or arousal levels because of the medication they were taking. Therefore, it is important to be mindful of such parameters when interpreting the results. Considering that some diseases are more prevalent in specific sex, sample size should be sex-matched to avoid selection bias. Some patients, such as vestibular patients, could also be less susceptible to any form of rehabilitation, possibly due the progressive physiological activity decline. Finally, some questionnaires are only validated in healthy younger adults and therefore should be validated in older adults and in individuals with specific diseases to establish the psychometric properties and thresholds before being used as reliable comparative measures. Unfortunately, there is still not enough evidence in the literature to provide a focused and critical review on the effects of HMD VR systems in a specific population or clinical condition. Still, this systematic review provides a comprehensive view on how HMD VR systems could be used to assess balance, and which are their potential benefits if used for training balance in older adults.

## Conclusions

HMD VR systems offer ecologically valid scenarios to assess and train functional balance that can be used alone or combined with other interventions. Various visual scenarios can be added, removed, isolated, and manipulated to identify and adjust to specific balance-related impairments of different clinical conditions. HMD VR training creates real-based scenarios that can be useful to improve postural control and gait patterns by altering motor planning and muscle activation. HMD VR systems could also augment fall prevention programs of those with higher risk of falling by providing more visual cues on how to handle context-based unexpected events. HMD systems can also be useful in identifying those with balance impairments, but there is still a need for further validation and cut-offs for each balance metric and adjustment to be made according to the pathological conditions. The risk of bias and overall quality of studies is still a major concern. There is a need of high-powered and high-quality studies before any definitive recommendations could be made on the use of HMD VR training to improve balance in older adults. Future studies should focus on establishing new norms and protocols that are adjusted to different individual characteristics, including age, gender, health status, type and severity of clinical condition, and cognitive and physical impairments, for maximizing their benefits.

## Author Contributions

PS designed the study, performed the database searches, extracted the data, analyzed the results, drafted the manuscript, and performed the risk of bias judgment. RA contributed to the analysis of the results, risk of bias assessment, drafting manuscript, and critical revision. Both authors approved the manuscript.

## Conflict of Interest

The authors declare that the research was conducted in the absence of any commercial or financial relationships that could be construed as a potential conflict of interest.

## Appendix

**Table:**

Database	Search strategy
PubMed and Web of Science	((HMD OR head mount^*^ display OR “virtual reality” OR “immersive reality” OR “artificial environment” OR “simulated 3D environment” OR “simulated three-dimensional environment”) AND (balance OR posture OR fall)) AND (vestibular OR visual OR somatosensory OR context^*^)
Scopus	((hmd OR head AND mount^*^ AND display OR “virtual reality” OR “immersive reality” OR “artificial environment” OR “simulated 3D environment” OR “simulated three-dimensional environment”) AND (balance OR posture OR fall)) AND (vestibular OR visual OR somatosensory OR context^*^) not INDEX (medline)
EBSCOhost	((HMD OR head mount^*^ display OR “virtual reality” OR “immersive reality” OR “artificial environment” OR “simulated 3D environment” OR “simulated three-dimensional environment”) AND (balance OR posture OR fall)) AND (vestibular OR visual OR somatosensory OR context^*^)
